# Mindfulness meditation styles differently modulate source-level MEG microstate dynamics and complexity

**DOI:** 10.3389/fnins.2024.1295615

**Published:** 2024-02-02

**Authors:** Antea D’Andrea, Pierpaolo Croce, Jordan O’Byrne, Karim Jerbi, Annalisa Pascarella, Antonino Raffone, Vittorio Pizzella, Laura Marzetti

**Affiliations:** ^1^Department of Neuroscience, Imaging and Clinical Sciences, University of Chieti-Pescara, Chieti, Abruzzo, Italy; ^2^Department of Psychology, University of Montreal, Montreal, QC, Canada; ^3^Institute for the Applications of Calculus “M. Picone”, National Research Council, Rome, Lazio, Italy; ^4^Department of Psychology, Sapienza University of Rome, Rome, Lazio, Italy; ^5^Institute for Advanced Biomedical Technologies, University of Chieti-Pescara, Chieti, Abruzzo, Italy

**Keywords:** microstate analysis, brain criticality, complexity, mindfulness meditation, open monitoring meditation, focused attention meditation, magnetoencephalography

## Abstract

**Background:**

The investigation of mindfulness meditation practice, classically divided into focused attention meditation (FAM), and open monitoring meditation (OMM) styles, has seen a long tradition of theoretical, affective, neurophysiological and clinical studies. In particular, the high temporal resolution of magnetoencephalography (MEG) or electroencephalography (EEG) has been exploited to fill the gap between the personal experience of meditation practice and its neural correlates. Mounting evidence, in fact, shows that human brain activity is highly dynamic, transiting between different brain states (microstates). In this study, we aimed at exploring MEG microstates at source-level during FAM, OMM and in the resting state, as well as the complexity and criticality of dynamic transitions between microstates.

**Methods:**

Ten right-handed Theravada Buddhist monks with a meditative expertise of minimum 2,265 h participated in the experiment. MEG data were acquired during a randomized block design task (6 min FAM, 6 min OMM, with each meditative block preceded and followed by 3 min resting state). Source reconstruction was performed using eLORETA on individual cortical space, and then parcellated according to the Human Connect Project atlas. Microstate analysis was then applied to parcel level signals in order to derive microstate topographies and indices. In addition, from microstate sequences, the Hurst exponent and the Lempel-Ziv complexity (LZC) were computed.

**Results:**

Our results show that the coverage and occurrence of specific microstates are modulated either by being in a meditative state or by performing a specific meditation style. Hurst exponent values in both meditation conditions are reduced with respect to the value observed during rest, LZC shows significant differences between OMM, FAM, and REST, with a progressive increase from REST to FAM to OMM.

**Discussion:**

Importantly, we report changes in brain criticality indices during meditation and between meditation styles, in line with a state-like effect of meditation on cognitive performance. In line with previous reports, we suggest that the change in cognitive state experienced in meditation is paralleled by a shift with respect to critical points in brain dynamics.

## 1 Introduction

The investigation of mindfulness meditation practice, defined as a non-judgmental awareness training leading to several modifications in cognitive and affective processes (Cahn and Polich, 2006; Lutz et al., 2008), has seen a long tradition of theoretical, affective, neurophysiological and clinical studies. Specifically, mindfulness meditation has been classically divided into two main styles: focused attention meditation (FAM), in which sustained attention is focused on a defined meditative object (e.g., breath), and open monitoring meditation (OMM) which represents the ability to experience the environment and mind–body processes in a non-reactive and non-judgmental manner (Lutz et al., 2008). With reference to the taxonomy of Dahl et al. (2015), FAM is part of the attentional family of practices, a class of meditation practices that strengthen the self-regulation of various attentional processes, by involving a narrowing of attentional scope; while OMM practice, in our study, involves facets of the attentional family, in terms of releasing attentional control and bringing awareness to whatever enters the field of consciousness, but also implicates facets of the deconstructive family, i.e., insights into the processes of perception, emotion, and cognition.

The study of neurophysiological and neurobiological bases underpinning meditation practice is gaining momentum due to the relevance of meditation-based interventions in cognitive-behavioral therapy, to treat several mental disorders (Hofmann and Gómez, 2017; Apolinário-Hagen et al., 2020). To date, the investigation of neural correlates shaped by, and supporting, meditation processes has seen a rapid increase. Indeed, neuroimaging studies have highlighted the brain structures and functional networks that play a key role in the orchestration of meditation practice (Lutz et al., 2008; Raffone and Srinivasan, 2010; Marzetti et al., 2014; Guidotti et al., 2023).

In particular, the high temporal resolution of electrophysiological neuroimaging techniques such as magnetoencephalography (MEG) or electroencephalography (EEG), has widely been exploited to fill the gap between the personal experience of meditation practice and brain activity subserving modifications in consciousness (Lutz and Thompson, 2003), allowing the investigation of the rapidly changing dynamics characterizing the interaction between different brain regions involved in the meditation practices (Deolindo et al., 2020). Mounting evidence, in fact, shows that human brain activity is highly dynamic and non-stationary, transiting between different brain states coding for a wide range of cognitive functions (Brodbeck et al., 2012; Britz et al., 2014; Milz et al., 2016; Seitzman et al., 2017; Liégeois et al., 2019; Zappasodi et al., 2019; Zhou et al., 2019). Additionally, the temporal dynamic of these brain-states has been identified as a possible neurophysiological signature of abnormal self experience in clinical populations (Vellante et al., 2020), suggesting that the study of the brain-states activity could allow the investigation of early endophenotypes in genetic condition (i.e., 22q11.2) influencing the onset of schizophrenia (Tomescu et al., 2015; Piccini et al., 2017).

Distinct brain states are associated with specific patterns of synchronized activity within and across brain regions (Baker et al., 2014; Michel and Koenig, 2018; Vidaurre et al., 2018; Tait and Zhang, 2022b). One widely used approach for the identification of brain states, known as EEG microstate analysis (Khanna et al., 2014; Michel and Koenig, 2018), provides a data-driven temporal clustering of topographical configurations of the synchronized activity, avoiding an arbitrary *a priori* definition of time windows of interest (Murray et al., 2008; Croce et al., 2020). These configurations can in turn be described by several metrics (e.g., occurrence, variance, duration, etc.). In addition, the complexity and criticality of dynamic transitions between microstates have been proposed as indices to differentiate between patient populations with different levels of cognitive impairment (Tait et al., 2020), or between levels of consciousness, such as wakefulness and sleep (Von Wegner et al., 2023), while it has never been explored in meditation. Criticality refers to the delicate balance between order and disorder in the brain’s electrical activity, a state where emergent long-range correlations endow brain dynamics with both stability and flexibility, crucial for optimal cognitive functioning. As such, deviations from criticality have been linked to altered states of consciousness (O’Byrne and Jerbi, 2022). Also, the relation between brain criticality and meditation is still at an early stage (Irrmischer et al., 2018; Dürschmid et al., 2020; Walter and Hinterberger, 2022). Even though EEG microstate analysis provides robust and reproducible results, conventional EEG microstate analysis entails certain limitations in terms of functional and anatomical interpretation, due to the clustering being performed from sensor-space data. To overcome these limitations, the present study relies on data from magnetoencephalography (MEG) after source-space projection to investigate microstate dynamics (Tait and Zhang, 2022b) and brain criticality in mindfulness meditation with high temporal and spatial resolution. Grounding our investigation on the hypothesis that the brain, which is thought to operate near the edge of a critical phase transition between order and disorder (O’Byrne and Jerbi, 2022), may reduce its distance to the critical point when switching to a controlled meditative condition, we relied on data from a group of long-term meditators with an outstanding expertise in both FAM and OMM, i.e., Theravada Buddhist monks. Our goal was twofold: we aimed to (i) derive source-level microstates, and (ii) investigate modulations of microstate dynamics across the different meditation practices, specifically in relation to avalanche criticality and edge-of-chaos phase transitions.

## 2 Materials and methods

### 2.1 Participants and procedures

Ten right-handed Theravada Buddhist monks (all males, mean age 38.7 years, range 25.0–58.0 years, SD 10.9 years), recruited from the Santacittarama Monastery, in central Italy, with a meditative expertise of minimum 2,375 h (mean meditation hours 14,765, range 2,375–26,600, SD 8018), participated in the experiment. Monks from the Santacittarama Monastery follow a Theravada Thai Forest Tradition, in which monks experience regular intensive meditation retreats, with a balanced practice of Focused Attention Meditation - FAM (Samatha) and Open Monitoring Meditation – OMM (Vipassana), including an about 3-month long winter retreat. Outside the retreat period, the monks typically practice Samatha–Vipassana meditation, with a balance of FAM and OMM meditation, 2 h per day with the monastery community. Individual meditation practice, with a balance of FAM and OMM is also emphasized.

**Figure 1:**
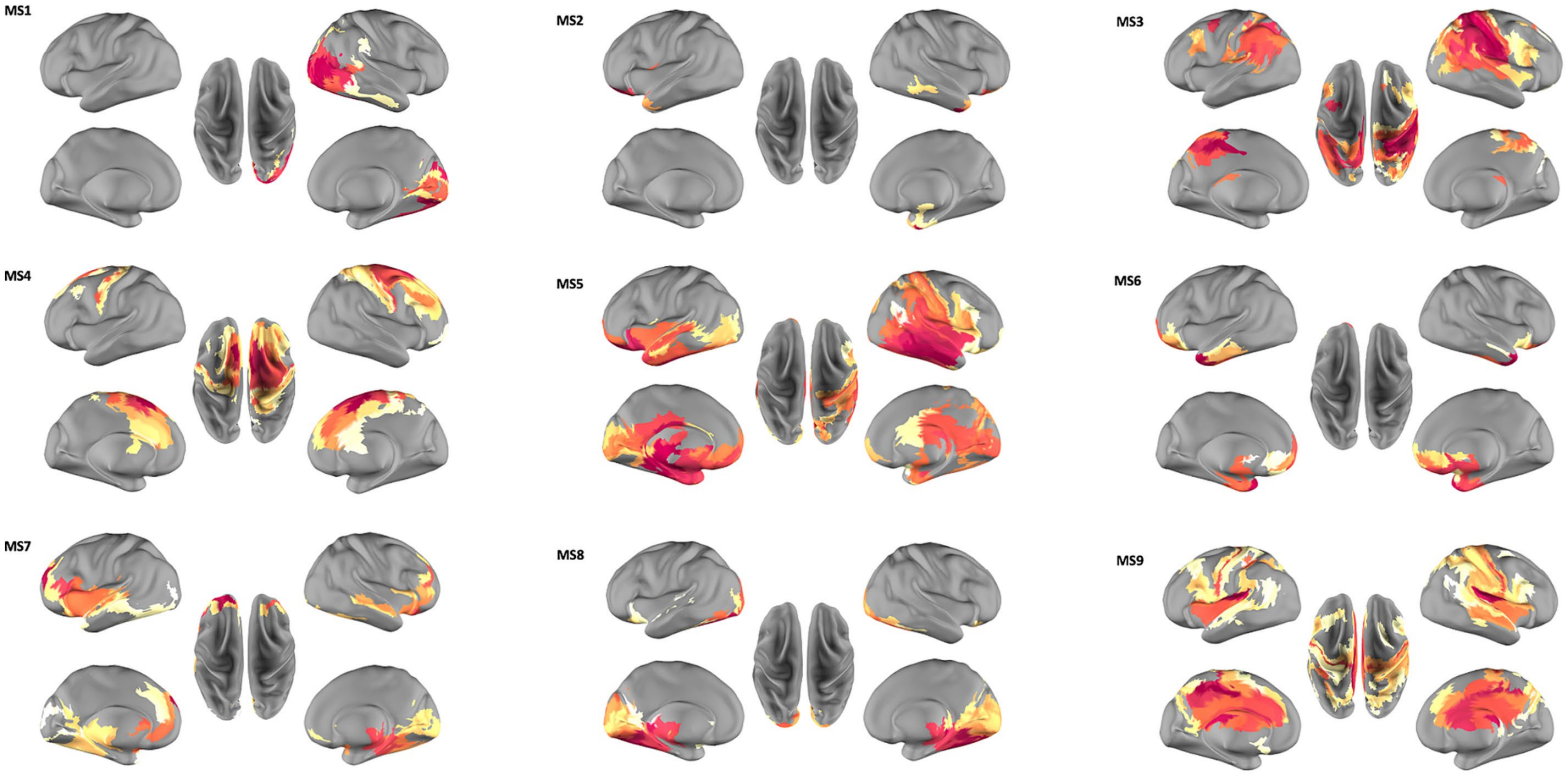
Spatial organization of the nine microstates (MS) extracted from source space MEG data. MS1, right-lateralized visual areas; MS2, orbital fronto-polar and lateral-temporal areas; MS3, parietal cortices and dorsolateral prefrontal cortices; MS4, middle cingulate-frontal system; MS5, temporo-parietal junction, the ventral visual system and a right lateralized fronto-parietal circuit; MS6 and MS7, fronto-temporal activity; MS8, temporo-occipital areas; MS9, prefrontal cortices, cingulate cortex and parietal areas. A more detailed list of parcels represented in each microstate is provided in Supplementary Figure S2.

All participants underwent standard screening procedures for MEG and structural Magnetic Resonance Imaging (MRI). The experiment was conducted with the subject written informed consent according to the Declaration of Helsinki, as well as with the approval of the local responsible Ethical Committee. MEG data were acquired during a randomized block designed experimental task, in which participants were asked to perform 6 min of FAM and 6 min of OMM; each meditative block was preceded and followed by 3 min of resting state (REST). All meditation and rest conditions were performed with eyes open. Before the experiment started, participants were given specific instructions (see Supplementary material) on how to perform the different meditation styles and, prior to the beginning of each recording block, the meditative style to be practiced was instructed by an experimenter through an auditory word-signal consisting in the condition name. The same data have been used in (Marzetti et al., 2014), see Figure 1 therein for a schematic of an exemplary sequence within the task protocol.

### 2.2 MEG data acquisition and preprocessing

MEG recordings were performed inside a magnetically shielded room at the Institute for Advanced Biomedical Technologies (ITAB), University of Chieti-Pescara (Pizzella et al., 2001; Chella et al., 2012) using a 165-channel MEG system, composed by 153 integrated dc SQUID magnetometers arranged on a helmet covering the whole head and 12 reference channels. Electro-cardiogram (ECG) and electro-oculogram (EOG) signals were also recorded for artifact rejection and all signals were band-pass filtered at 0.16–250 Hz and digitized at 1 kHz. Participant’s head position was recorded, acquiring the signal from five coils placed on the subject’s scalp, after at the end of each meditation practice run or at the end of the REST run. In order to allow co-registration to anatomical magnetic resonance images, the coil positions and anatomical landmarks (left and right preauricular and nasion) were measured by means of a 3D digitizer (3Space Fastrak; Polhemus) yielding the definition of a subject specific coordinates system. Magnetic resonance images were acquired using a sagittal magnetization prepared rapid acquisition gradient echo T1-weighted sequence (MP-RAGE; Siemens Vision scanner 1.5 T; TR = 9.7 s, echotime TE = 4 ms, alpha = 12°, inversion-time = 1,200 ms, voxel-size = 1 × 1 × 1 mm^3^).

The recorded MEG data were first band-pass filtered in the range 1–150 Hz using a cascade of Chebyshev Type II filters (high-pass: order 5; low-pass: order 24) available in Matlab (Mathworks) and then pre-processed by using an independent components analysis based algorithm (Mantini et al., 2011). In brief, the algorithm projects the MEG data onto a set of maximally independent components and automatically classifies them, thus identifying artifactual components (e.g., cardiac artifact, eye movements) and components generated by brain signals. A similar classification procedure has also been employed in Saggar (2011). Finally, sensor level cleaned data are obtained by subtracting components labeled as artifactual from the raw signals.

### 2.3 Microstate analysis in source space

Microstate analysis is usually applied to extract brain dynamics from sensor space data. Such an approach reduces the EEG/MEG time course to a sequence of states, each represented by a specific scalp topography. Scalp topographies are usually extracted by clustering the EEG/MEG signal in sensor space using algorithms such as k-means or hierarchical clustering (Khanna et al., 2015; von Wegner and Laufs, 2018).

Here, we utilize an alternative approach, proposed in Tait and Zhang (2022b), to compute microstates directly from source-reconstructed MEG data. Specifically, the source-level MEG microstate analysis pipeline consists of the following steps: MEG data preprocessing (described in the previous paragraph), MEG source signal reconstruction, clustering of source-level topographies, backfitting and calculation of (i) microstate metrics, (ii) criticality indices for microstate transitions. These steps, but for preprocessing, are described below.

#### 2.3.1 Source reconstruction

Source reconstruction was performed using the eLORETA approach (Pascual-Marqui, 2007) on individual cortical space implemented in Fieldtrip (Oostenveld et al., 2011). The source reconstruction pipeline started, using the Freesurfer software (Fischl, 2012), by extracting, from the T1-weighted MRI image, the scalp, brain, and cortical surfaces. Cortical surfaces were labeled according to the HCP230 atlas (Tait et al., 2021). The cortical mesh was downsampled to approximately 10,000 vertices to generate a set of dipole locations to be used as source space using the ‘iso2mesh’ software (Fang and Boas, 2009) and dipoles were oriented normally to the cortical surface (Dale et al., 2000; Hillebrand and Barnes, 2003). Fieldtrip (Oostenveld et al., 2011) was used to build an individual single shell volume conductor model (Nolte, 2003) for MEG forward problem solution. Parcel time courses were then band-pass filtered in the 1–30 Hz frequency band in line with sensor-level EEG microstate studies (Michel and Koenig, 2018). Since source reconstruction approaches usually use several thousand of cortical vertices to compute distributed sources, here, as suggested in Tait and Zhang (2022b), the cortical mantle was parcellated into regions of interest obtained using the HCP230 atlas (Tait et al., 2021), a version of the Human Connectome Project’s multimodal parcellation (Glasser et al., 2016) optimized for resting-state MEG. Vertices were thus associated with the different parcels and parcel-level signals were obtained from the first principal component of all voxel time courses within a parcel. This approach allowed us to derive 230 parcel time courses to be used for further analysis.

#### 2.3.2 Clustering of source level topographies

In order to extract microstate class templates, the modified version of the k-means clustering algorithm proposed in Tait and Zhang (2022b) was employed, using the code in Tait and Zhang (2022a). In brief, such a clustering algorithm differs from the classical k-means algorithm in that, in source space, new cluster centroids are calculated as the eigenvector corresponding to the largest eigenvalue of the matrix representing the maps within a specific cluster. In this way, this eigenvector is equal to the first principal component in the case of zero mean data. Details are reported in Tait and Zhang (2022b). Only the samples corresponding to maxima of Global Field Power (GFP) were used as input to the clustering algorithm. In source-reconstructed data, the GFP is calculated as the vector norm of the parcel signals which correspond to the total deviation from a zero current density. As suggested in Tait and Zhang (2022b), 5,000 GFP peaks from each subject and from each condition (REST, FAM, OMM) were extracted. Hence, across subjects and conditions, a total of 150,000 GFP peaks (5,000 GFP peaks × 10 subjects × 3 conditions) were submitted to the clustering k-means algorithm, and the microstate class maps were obtained. In order to set the optimal number of microstates, all GFP peaks extracted from data from all conditions (REST, FAM, OMM) were submitted to the *k*-means clustering algorithm varying the number of states (*k*) from 2 to 20. The kneedle algorithm (Tait and Zhang, 2022b) was used to establish the optimal number of states. Indeed, in clustering, the knee represents the point at which adding further clustering fails to add significantly more detail. In our case the kneedle algorithm suggested *k* = 9 as the optimal number of states.

#### 2.3.3 Backfitting and estimation of microstate metrics

Through the backfitting procedure, MEG time series are reduced to a sequence of states. The presence of each state is identified by assigning each instantaneous source space topography to one of the previously identified microstate classes on the basis of the spatial correlation between the instantaneous topography and the microstate class maps (Koenig et al., 1999; Michel and Koenig, 2018). Once the sequence of states has been identified, several spatiotemporal and criticality metrics can be calculated. Here, we relied on the Microstate+ toolbox (Tait and Zhang, 2022a) to compute microstate-specific and global metrics: microstate Duration, Coverage, and Occurrence, as well as Hurst exponent and Lempel-Ziv complexity of microstate sequences. These indices are defined in the different conditions (REST, FAM, OMM) for each microstate and for each participant.

Specifically, the Duration of a given microstate is defined as the average of the time covered by the microstate and can be interpreted as a measure of microstate stability. Microstate Coverage is the percentage of time spent in a single microstate class representing a measure of dominance. The Occurrence of a microstate is computed as the number of times a given state occurs over the whole registration divided by the duration of the registration. Note that Occurrence, Coverage and Duration are interrelated, according to the following relationship Coverage = (normalized) Duration × Occurrence.

Criticality is often indicated by the presence of long-range temporal correlations. This temporal dependency -also known as signal memory- can be quantified using the Hurst exponent H (Beggs, 2022; O’Byrne and Jerbi, 2022). Indeed, an Hurst exponent that deviates from the 0.5 exhibits also fractal-like properties (self-similarity). Therefore our results for the Hurst exponent can be related to previous EEG studies (Zappasodi et al., 2014, 2015; Porcaro et al., 2022).

For 0.5 < *H* < 1, long-range dependency is observed, where the occurrence of a given microstate makes future occurrences of that microstate more likely; conversely, for 0 < *H* < 0.5, there is also long-range memory, but it is anticorrelated, meaning that the occurrence of a microstate makes future occurrences of that microstate less likely. As *H* approaches 0.5, the signal becomes increasingly uncorrelated in time, with *H* = 0.5 indicating a memoryless signal (Hardstone et al., 2012; Palva et al., 2013). Here, we use this index, calculated as in (Tait and Zhang, 2022a) to study long-range temporal correlation, or temporal self-affinity, in the microstate sequences.

Specifically, the Hurst exponent was calculated through Detrended Fluctuation Analysis (DFA; Croce et al., 2018). To perform this analysis, the sequence of microstates needs to be embedded into a random walk. We constructed the random walk modifying the procedure used in (Van de Ville et al. 2010). The microstate sequence was randomly partitioned into two classes assigning the values −1 and 1 to each class (e.g., for 6 microstates we may obtain for a given repetition C1 = {1, 4, 6}, C2 = {2, 3, 5}). In the present case of an odd number N of microstates, to avoid class imbalance, a “leave-one-out” procedure was used. Specifically, we left out a randomly chosen microstate from the sequence and created a bipartition of the remaining N-1 microstates. One thousand repetitions of the random partitioning was performed, the DFA for each repetition was calculated, and the final DFA value was taken as the average across repetitions.

In detail, for each repetition, the DFA is calculated as follows. The random walk process is calculated as the cumulative sum of the embedded microstate sequence. Subsequently, the cumulative sum was divided into Ns segments of size $s=2^{n}$ with n varying from 6 ( $2^{6}=64$ as a minimum reliable estimate) to maximum n, which would fit in the length of the random walk. For each segment, the local trend was determined using a least-square line fitting technique (Kantelhardt et al., 2001). Considering $X_{j,s}$ $\left(\left(i\right)\right)$ the ordinate of the fitting line of the $j_{th}$ segment of length s at time bin *i* (*i* = 1, 2, …*s*) the fluctuation of the $j_{th}$ segment of length *s*, i.e., the root-mean-square deviation from the trend, was calculated as:


$$RMS_{j}^{s}=\frac{1}{2}\overset{s}{\underset{i=1}{\sum }}{\left\{x\left[\left(j-1\right)s+i\right]-x_{j,s}\left(i\right)\right\}}^{2}$$

To derive the fluctuation function, the average of the root mean square deviation from the trend was computed for each scale s, following the approach outlined by Kantelhardt et al. (2001):


$$F\left(s\right)=\sqrt{\frac{1}{N_{s}}\overset{S}{\underset{j=1}{\sum }}RMS_{j}^{s}}$$

The scaling characteristics of the fluctuation function can be revealed through a logarithmic plot of F(s) against s. If there is a presence of long-range power-law correlation, the subsequent relationship holds:

*F*(*s*) ∼ *sH.*

and the plot is a line, with slope equal to H, the Hurst exponent (Feder, 1988).

Finally, the Lempel-Ziv complexity (Lempel and Ziv, 1976) is a measure of the edge of chaos phase transitions (O’Byrne and Jerbi, 2022) indexing complexity and is inversely related to the compressibility of a string of symbols (the temporal sequence of microstates in our case). Such a measure is based on the idea that the more repetitive patterns there are in a string, the less complex and the more compressible it is. Specifically, a string is considered to possess low LZC when it contains only a few frequently recurring sequences. This implies that the string can be compressed into a small data size. Microstate sequences characterized by low LZC exhibit redundancy, involving a constrained set of transitioning patterns within the sequence. Conversely, high LZC indicates intricate and diverse transitioning patterns, suggesting complexity. The procedure for calculating the Lempel-Ziv complexity from the microstate sequence is as follows. The microstate sequence, which might contain consecutive occurrences of the same microstate (e.g., 4 microstates A,B, C, D appearing in the following sequence BBAAADAADDCCC), is transformed into a transitioning sequence. This transitioning sequence captures the transitions between microstates. In the above example, BBAAADAADDCCC is reduced to BADADC. The decision to calculate LZC based on the transitioning sequence, rather than on the raw microstate sequence, was influenced by the observation that the raw microstate sequence is significantly linked to the deceleration of neuronal oscillations, whereas the transitioning sequence lacks this strong correlation (Tait et al., 2020). Of note, Hurst exponent and Lempel-Ziv complexity are brain criticality measures which are not microstate-specific. Indeed, they are calculated from the temporal sequences of microstates in the different conditions for each participant (see Supplementary Figure S1 for a schematic representation of this procedure).

#### 2.3.4 Statistical analysis

All statistical analyses were performed using the Jamovi 2.3.18 software.

To assess differences in microstate metrics between the different conditions (REST, FAM, OMM), repeated measure Analysis of Variance (ANOVAs) were separately performed for each microstate metric (Duration, Occurrence, Coverage). A 9 × 3 design was applied, with Microstate Class and Condition (REST, FAM, OMM) as within-subject factors. *Post hoc* tests were performed to identify differences between metrics across conditions. Tukey’s HSD test was used to correct for multiple comparisons.

A three-level (REST, FAM, OMM) repeated measures ANOVA was performed to assess differences in the criticality indexes (Hurst exponent and Lempel-Ziv complexity) obtained from microstate temporal sequences. *Post hoc* tests were performed to identify differences between metrics across conditions. Tukey’s HSD test was used to correct for multiple comparisons.

Finally, we performed correlational analyses between meditation expertise (in years) of the 10 participants and individual differences of microstates metrics (Duration, Occurrence, Coverage) across conditions (REST, FAM, OMM) for each of the 9 microstates, as well as individual difference across conditions of criticality indices (Hurst exponent and Lempel-Ziv complexity) in order to understand whether a relation with expertise is present.

## 3 Results

### 3.1 Microstate topographies

From the clustering algorithm nine source reconstructed microstate maps were obtained, representing the spatial organization of each microstate (MS).

Figure 1 shows these nine topographical maps, labeled from 1 to 9 and each representing one Microstate Class with a specific pattern of synchronized brain activity. All maps show a bilateral pattern except for MS1, which exhibits a right-lateralized visual synchronized activity. MS2 pattern includes orbital fronto-polar and lateral-temporal areas; MS3 reveals a prominent synchronized activation of the parietal cortices and dorsolateral prefrontal cortices, both strongly involved in the Central Executive Network (CEN) control; while the MS4 map captures the activity of a middle cingulate-frontal system compatible with the Salience network. The pattern of synchronized activity exhibited by MS5 involves ventral attentional areas such as the temporo-parietal junction, the ventral visual system and a right lateralized fronto-parietal circuit; while MS6 and MS7 exhibit a prominent fronto-temporal activity, and MS8 shows a dominant pattern of temporo-occipital synchronization. MS6 and MS8 also include medial areas in the default mode system. Finally, MS9 exhibits a more extended pattern spanning from prefrontal cortices, cingulate cortex and parietal areas.

Details on parcels active in each microstate class are given in Table 1.

**Table 1:** List of ROIs included in the spatial topography of the different Microstates.

***MICROSTATES***									
	***MS1***	***MS2***	***MS3***	***MS4***	***MS5***	***MS6***	***MS7***	***MS8***	***MS9***
***ROIs***	Visual	Orbital polar frontal	Para central mid-cingulate	Supplementary and cingulate eye field	Insula frontal opercular	Medial temporal	Posterior opercular	Visual	ParaCentral_Mid-Cingulate
	Ventral visual complex	Lateral temporal	Superior parietal	ParaCentral_Mid-Cingulate	Medial temporal	Anterior cingulate medial PFC	Auditory	Ventral visual complex	Posterior opercular
	MT complex		Posterior parietal	Dorsolateral prefrontal	Posterior cingulate	Orbital polar frontal	Insula frontal opercular	MT complex	Auditory
	TPJ		Somatosensory	Premotor	Anterior cingulate medial PFC	Lateral temporal	Anterior cingulate medial PFC	Medial temporal	Posterior cingulate
	Inferior parietal		Auditory	Anterior cingulate medial PFC	Visual		Inferior parietal		Somatosensory
			Insula frontal opercular	Superior frontal language	Ventral visual complex		Dorsolateral PFC		Superior parietal
			TPJ	Right FEF	MT complex				Anterior cingulate medial PFC
			FEF		Somatosensory				Posterior parietal
			Perisilvian		Posterior opercular				Anterior cingulate medial PFC
			Dorsolateral PFC		Lateral temporal				
					Perisilvian language				
					TPJ				
					Inferior parietal				
					Inferior frontal				
					Auditory				

The list is defined on the basis of cortical areas in the parcellation provided by the HCPex atlas (Huang et al., 2022). Nomenclature is as follows: MT, middle temporal; TPJ, TemporoParietal Junction; FEF, frontal eye field; PFC, prefrontal cortex.

### 3.2 Condition specific differences In microstate-specific metrics

ANOVA and *post hoc* (paired *t*-test) results for each microstate-specific metric (Duration, Coverage, Occurrence) are provided in the following.

#### 3.2.1 Microstate duration

**Figure 2:**
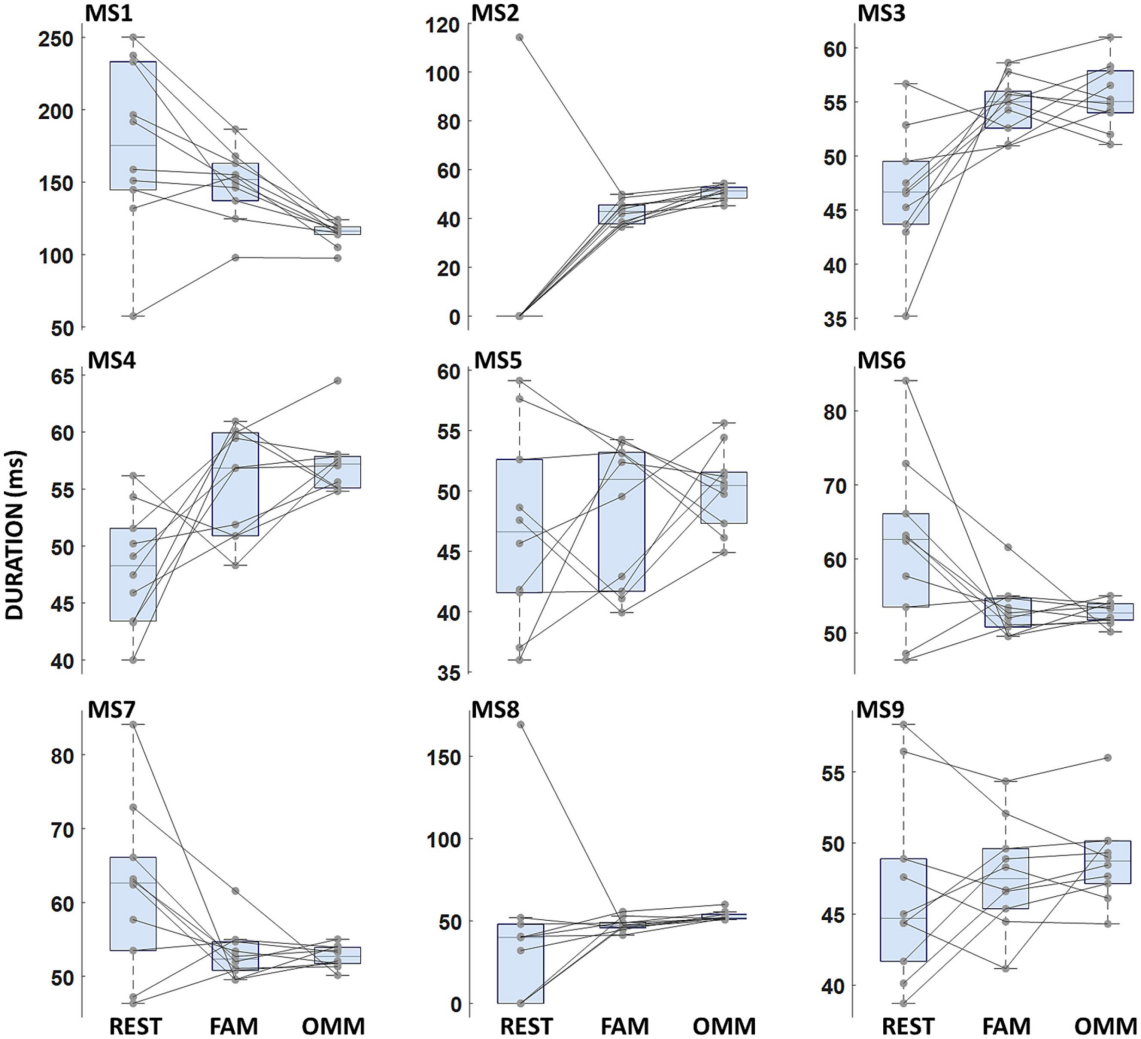
Microstate duration. Boxplots of microstate duration for each microstate. No significant differences between conditions were found.

Duration values for all microstates and conditions are reported in Figure 2. No significant Condition effect or interaction Microstate Class × Condition (REST, FAM, OMM) was found [*F*(2, 18) = 0.647; *p* = 0.535].

#### 3.2.2 Microstate coverage

**Figure 3:**
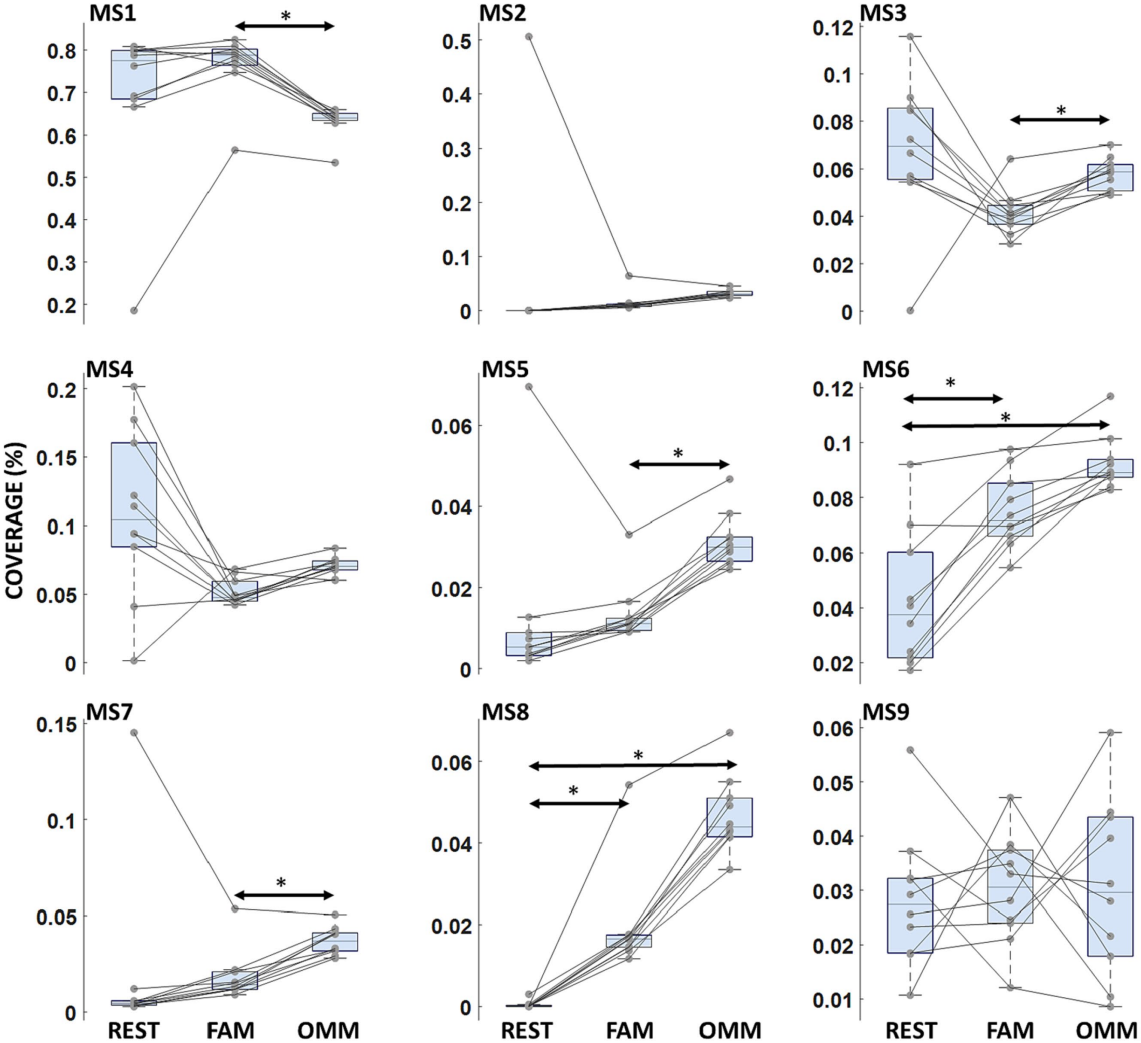
Microstate coverage. Boxplots of microstate Coverage for each microstate. Arrows indicate a significant modulation with the Condition factor. Horizontal bars indicate median values and whiskers mark the range from 25th to 75th percentile. Asterisks indicate statistically significant differences (*p* < 0.05, Tukey’s HSD corrected).

A Condition effect indicating differences in mean values of Coverage across conditions (REST, FAM, OMM) was found [*F*(2, 18) = 15.13; *p* < 0.001]. Moreover, a significant interaction Microstate Class x Condition (REST, FAM, OMM) was observed [*F*(7.0, 16) = 4.333; *p* < 0.001]. *t*-test *post hoc* comparison showed a significant decreasing of Coverage in OMM practice with respect to FAM practice for MS1 [*t*(9) = 9.7205; *p* < 0.001] and an increased Coverage in OMM practice with respect to FAM practice for MS5 [*t*(9) = −12.32; *p* < 0.001]. MS3, MS7 and MS8 showed an increasing progression of Coverage values from REST to FAM and to OMM [*t*(9) = −5.81; *p* = 0.019, *t*(9) = −6.10; *p* = 0.032, *t*(9) = −6.90; *p* = 0.012]. For MS6 and MS8, Coverage in the REST condition was significantly lower than in the OMM condition [*t*(9) = −6.05; *p* < 0.014, *t*(9) = −18.05; *p* < 0.001] and Coverage in the FAM condition was significantly lower than in the OMM condition [*t*(9) = −7.52; *p* = 0.003, *t*(9) = −12.92; *p* < 0.001]. Figure 3 shows the Coverage results across conditions where the significantly modulating microstates are marked.

#### 3.2.3 Microstate occurrence

**Figure 4:**
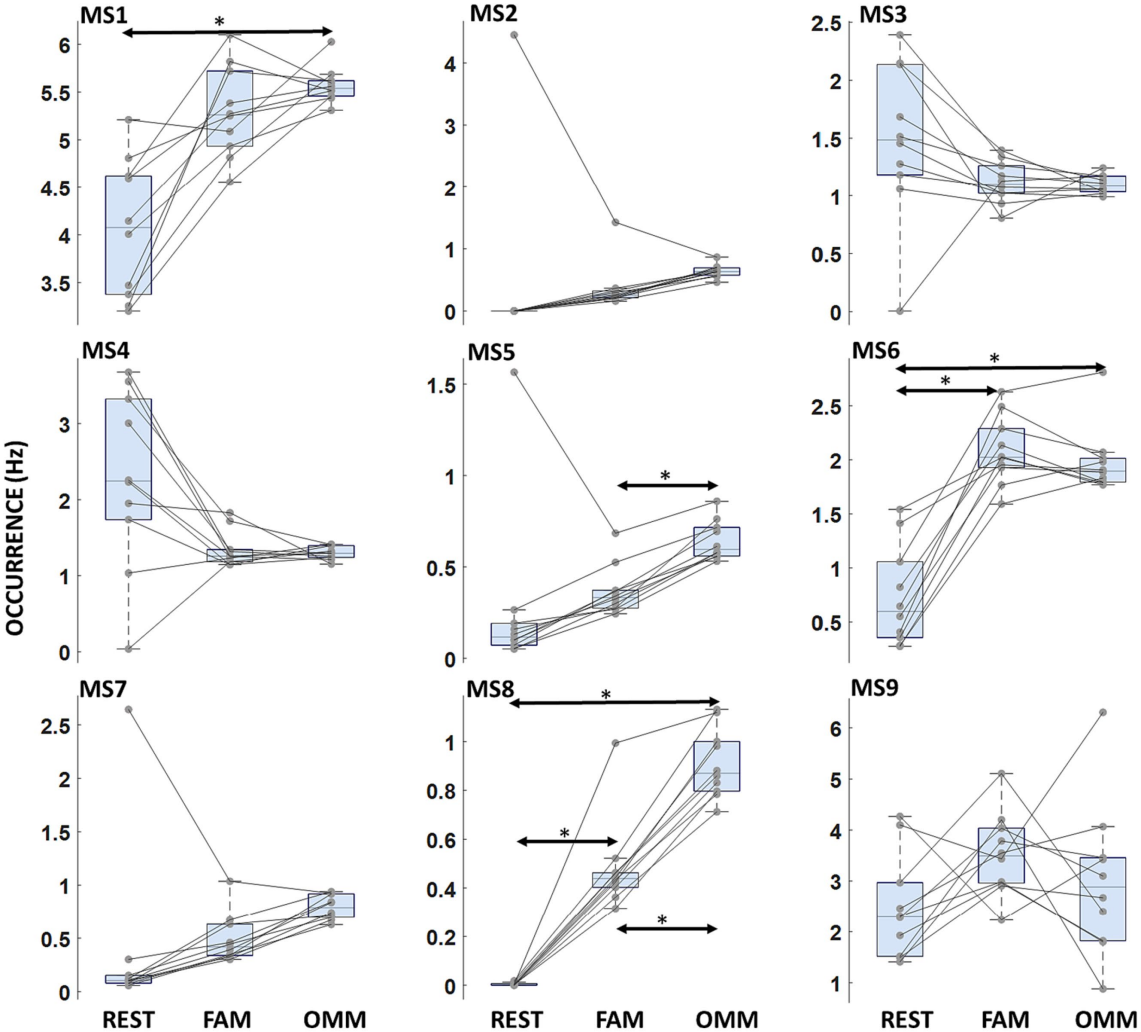
Microstate occurrence. Boxplots of microstate Occurrence for each microstate. Arrows indicate a significant modulation with the Condition factor. Horizontal bars indicate median values and whiskers mark the range from 25th to 75th percentile. Asterisks indicate statistically significant differences (*p* < 0.05, Tukey’s HSD corrected).

A Condition effect indicating differences in mean values of Occurrence across REST, FAM, and OMM conditions was found [*F*(2, 18) = 21.30; *p* < 0.001]. A significant interaction Microstate Class × Condition was found [*F*(7.0, 16) = 9.680; *p* < 0.001]. Post-hoc analysis showed a significantly increased Occurrence during OMM practice with respect to FAM for MS5 [*t*(9) = −10.47; *p* < 0.001]. MS8 showed an increasing progression of Occurrence values from REST to FAM and to OMM [*t*(9) = −8.94; *p* < 0.001, *t*(9) = −22.67; *p* < 0.001, respectively]. Conversely, MS6 showed a lower Occurrence in the REST condition with respect to both FAM and OMM practices [*t*(9) = −9.00; *p* = 0.001, *t*(9) = −6.85; *p* = 0.012]. No significant difference between FAM and OMM practices was found for the Occurrence of MS6. Figure 4 shows the Occurrence results where the significantly modulating microstates are marked.

#### 3.2.4 Microstate sequence criticality

**Figure 5:**
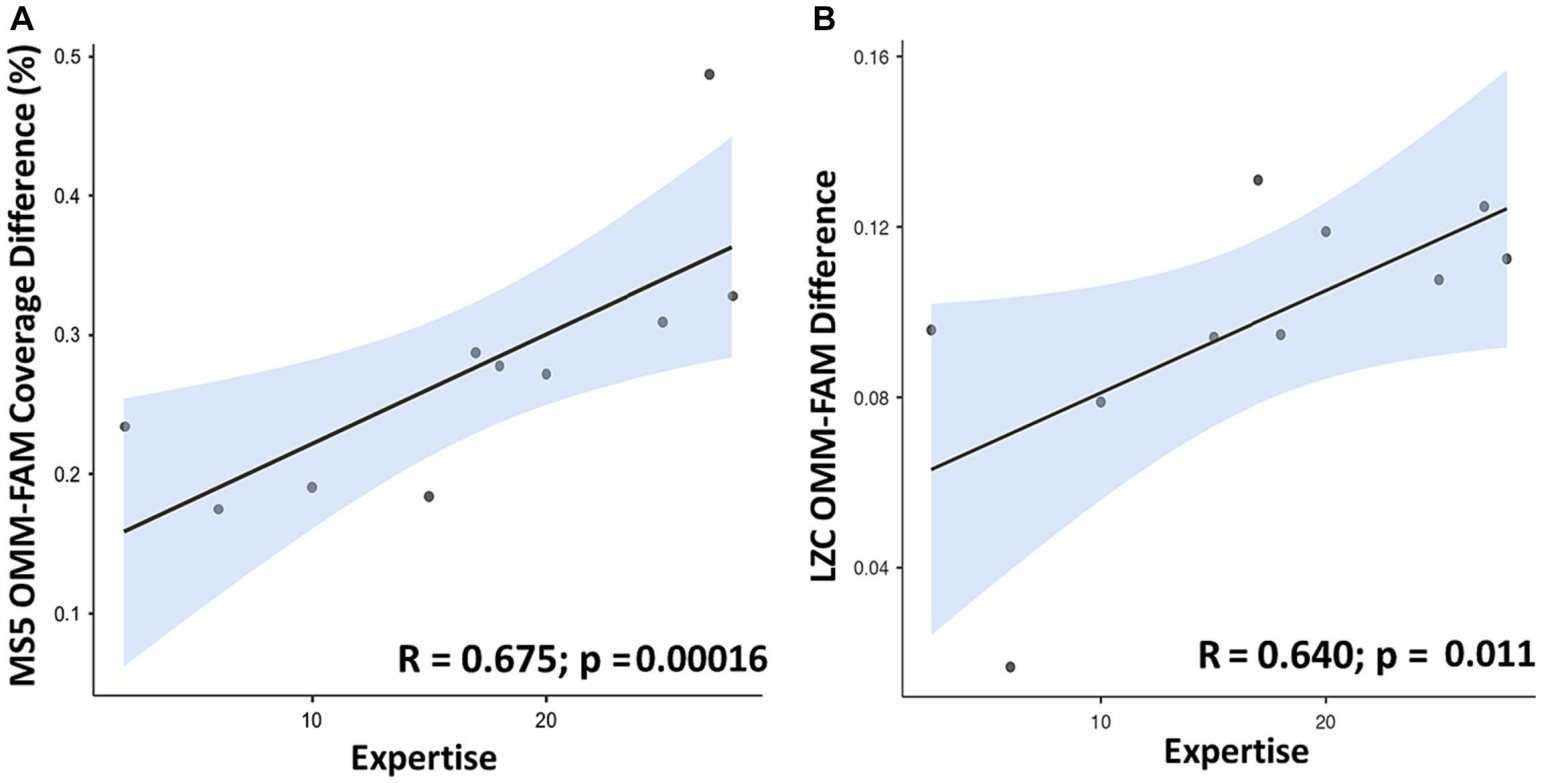
Criticality measures for microstate sequences in the different conditions. **(A)** Hurst exponent: boxplots refer to Hurst exponent values in the different conditions. **(B)** Lempel-Ziv complexity (LZC): boxplots refer to LZC values in the different conditions. In both panels, horizontal bars indicate median values and whiskers mark the range from 25th to 75th percentile. Asterisks indicate statistically significant differences (*p* < 0.05, Tukey’s HSD corrected).

For the Hurst exponent, a significant effect of Condition (REST, FAM, OMM) was found [*F*(2, 18) = 13.5, *p* = < 0.001]. *Post hoc* analysis showed that the Hurst exponent was higher in the REST condition with respect to both FAM and OMM conditions [*t*(9) = 4.15, *p* = 0.009; *t*(9) = 3.41, *p* = 0.025]. For the Lempel-Ziv complexity, a significant effect of Condition was found [*F*(2, 18) = 93.8; *p* < 0.001]. *Post hoc* analysis showed that Lempel-Ziv complexity changed in all conditions. In particular, an increasing trend in Lempel-Ziv complexity from REST to FAM and to OMM can be observed [*t*(9) = −5.40, *p* = 0.001; *t*(9) = −14.46, *p* < 0.001; *t*(9) = −9.46, *p* < 0.001]. Figures 5A,B shows the criticality results.

#### 3.2.5 Relation to meditation expertise

**Figure 6:**
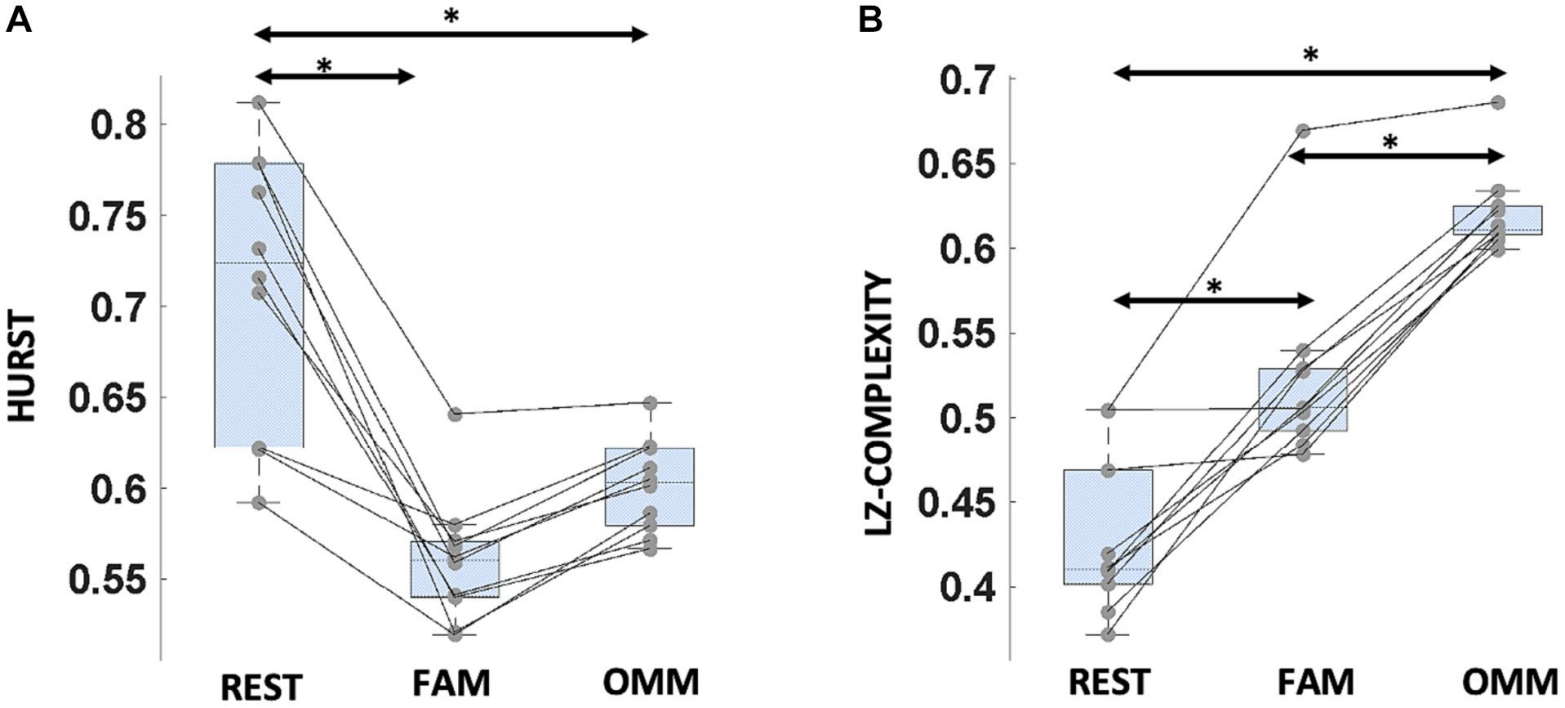
Scatterplots showing a positive linear association between meditation Expertise (expressed as years of meditation practice on the *x*-axis) and: **(A)** individual differences between values of MS5 Coverage (in %) in OMM and FAM conditions (OMM minus FAM); **(B)** individual difference between values of Lempel-Ziv complexity in OMM and FAM conditions (OMM minus FAM).

A positive linear association (*R* = 0.675; *p* < 0.00016 uncorrected) between meditation Expertise (expressed as years of meditative practice) and the individual differences in Coverage between OMM and FAM conditions for MS5 was observed (see Figure 6A). Coverage between OMM and REST and between FAM and REST, well as Duration and Occurrence for MS5 did not yield significant results. No significant correlation was observed for the other microstates.

For criticality indexes, a positive correlation (*R* = 0.640; *p* < 0.011 uncorrected) between meditation Expertise and the individual differences was observed between values of Lempel-Ziv complexity (LZC) in OMM and FAM, see Figure 6B. LZC complexity between OMM and REST and between FAM and REST did not significantly correlate with expertise. No significant correlation between the Hurst exponent and expertise was observed.

## 4 Discussion

In the present study, we show that meditation modulates the dynamics of selected microstates as well as the distance of the brain from the critical point measured from microstate sequences. More specifically, microstates featuring a high correspondence to visual, default mode and cingulo-opercular networks show a relatively higher presence and frequency during mindfulness meditation compared to rest; whereas microstate indexes differentially involved in the two meditation styles cover regions included in ventral attention, central executive, default, and somatomotor networks. Importantly, also the distance of the brain’s dynamical working point from the critical point appears to be modulated by the different conditions. More specifically, the Hurst exponent is higher during REST than in any of the meditative conditions, while the Lempel-Ziv complexity is lower in REST than in any of the meditative conditions but also lower in FAM in comparison to OMM, the difference between occurrence in OMM versus occurrence in FAM correlating with meditation expertise.

Although several efforts have been made in the understanding of brain modulations supported by mindfulness meditation practice, conventional M/EEG analyses may be not sufficient to explain the complex brain dynamics active during this process (Brandmeyer and Delorme, 2013). Therefore, we here rely on microstate analysis augmented by indices of brain criticality to investigate neural correlates of meditation practice. Our results for microstate indices show that, while the microstate duration is not significantly affected by the meditation style, the coverage and occurrence of specific microstates are modulated either by being in a meditative state or by performing a specific meditation style. In detail, MS6 and MS8 feature higher coverage and occurrence during meditative states compared to rest. These microstates mainly include regions from the Default Mode, Cingulo-opercular, and Visual networks and their greater involvement in meditation might be related to an increased awareness and regulation of higher mental imagery, spontaneous thoughts and mind wandering experienced during meditation (Xu et al., 2014; Tang et al., 2015; Panda et al., 2016).

Conversely, MS1, MS3, and MS5 feature a significant modulation between meditative states, with MS1, mainly including visual areas, more present in Focused Attention Meditation than in Open Monitoring Meditation, consistently with the FAM style possibly requiring higher focused attention which may also be intentionally oriented to an internal visualized target object (Fujino et al., 2018). Vice versa, MS3 and MS5, including regions from the Central Executive and Ventral Attention networks, are more present during Open Monitoring Meditation than during Focused Attention Meditation, possibly due to OMM practice requiring and improving larger attentional abilities and executive control resources (Tsai and Chou, 2016). In addition, differences in MS5 coverage between OMM and FAM positively correlate with individual expertise in meditation practice, i.e., monks with higher meditative expertise feature a larger difference in the coverage of MS5 during OMM versus FAM. MS5 involves regions of the Ventral Attention and Central Executive systems, of the Somatomotor network and of the Cingulo-opercular and Default Mode networks, thus the observed positive relation might be explained by an improved ability of experienced meditators to practice the more the more cognitively and metacognitively demanding OMM style while regulating mind wandering and emotions. It is worth noting that while MS3 and MS5 display considerable overlap with the Dorsal and Ventral Attention Networks as defined in fMRI studies (e.g., Thomas Yeo et al., 2011), other key resting state networks (RSNs) are only partially observed and can be distributed across multiple microstates. This partial representation could be attributed to a broader challenge observed in MEG resting state analysis, where various functional connectivity approaches have not fully reproduced the topographies of RSNs (e.g., de Pasquale et al., 2010; Brookes et al., 2011).

Importantly, our investigation aimed also at studying brain criticality changes from rest to different forms of mindfulness meditation. This investigation is grounded in the hypothesis that the brain, which is thought to operate near the edge of a critical phase transition between order and disorder (O’Byrne and Jerbi, 2022), may reduce its distance to the critical point when switching to a controlled meditative condition. Our cohort of highly experienced monk meditators represents an ideal model to test this hypothesis. Among critical phase transitions, we focused on avalanche and edge-of-chaos criticality as they have been shown to be particularly relevant to studying brain function and dysfunction (see O’Byrne and Jerbi, 2022 for a review). Specifically, we investigated changes in the Hurst exponent, as a measure of avalanche criticality, and in Lempel-Ziv complexity as a measure of edge-of-chaos criticality.

Our results indicate that in both meditation conditions, Hurst exponent values are reduced with respect to the value observed during rest, suggesting a reduced signal memory and a shift away from avalanche criticality during meditation.

These results are aligned with findings from Irrmischer et al. (2018) who showed a reduction of long-range temporal correlations of neural oscillations during FAM compared to REST in experienced meditators but not in meditation-naïve healthy volunteers, arguing that the focus of attention reduces information propagation by shifting the system toward a subcritical regime. Similarly, Walter and Hinterberger (2022) observed a significant reduction in long-range temporal correlation in three different meditation conditions (FAM, presence monitoring, thoughtless emptiness) compared to REST in highly proficient meditators.

Our results for Lempel-Ziv complexity show clear significant differences between OMM, FAM and REST, with a progressive increase in complexity from REST to FAM to OMM. An algorithmic interpretation of the Lempel-Ziv complexity is, in our case, that it essentially reflects how much a microstate sequence can be compressed. The progressive increase in complexity from REST to FAM to OMM can be interpreted as OMM being a more diverse state compared to FAM, and FAM a more diverse state compared to REST, possibly in line with the reduced cognitive demand from OMM to FAM and to REST (Raffone et al., 2019). In accordance, a previous study (Lu and Rodriguez-Larios, 2022) also showed a significant decrease in complexity, measured by Lempel-Ziv complexity and other metrics, during mind wandering as compared to breath focus states also in novices. In conjunction with recent work proposing that Lempel-Ziv complexity is maximized at the edge of chaos (Toker et al., 2022), these results suggest that during meditation, the brain shifts its operating point closer to the edge of chaos, with open monitoring meditation achieving the shortest distance to the critical point. Indeed, during OMM, meditators exhibit a maximal capacity to consciously process diverse information such as bodily sensations, feelings, and thoughts in line with the maximal information storage observed at the edge of chaos (Boedecker et al., 2012; Suárez et al., 2021). In addition, our results suggest that participants with less expertise may feature a reduced difference of Lempel-Ziv complexity, possibly indicating that greater expertise is needed to successfully perform OMM. Interestingly, being closer to the critical point might be instrumental for reaching non-reactive and non-judgmental awareness typical of Open Monitoring Meditation, which requires several years of practice. We note, however, that the relationship between Lempel-Ziv complexity and criticality remains somewhat unclear, with a recent report suggesting that Lempel-Ziv complexity of EEG microstate sequences is not maximized at criticality, but rather, continues to increase in the supercritical phase (von Wegner et al., 2023). Further numerical and analytical work will be needed to disambiguate this relationship. Moreover, other studies, using different metrics, support the findings of higher complexity during meditation (Kakumanu et al., 2018; Martínez Vivot et al., 2020). In conclusion, our investigation reports, for the first time to our knowledge, a source-space microstate analysis of magnetoencephalographic data in mindfulness meditation, with findings pointing toward relevant differences in the occurrence and coverage of microstates in the different conditions. Moreover, we report changes in brain criticality indices during meditation and between meditation styles, in line with a state-like effect of meditation on cognitive performance (Xu et al., 2014). Together with previous reports (Irrmischer et al., 2018), our results suggest that the change in cognitive state experienced in meditation is paralleled by a shift with respect to critical points in brain dynamics, supporting the relevance of the distance to criticality (and its control) for shifting between modes of cognition (O’Byrne and Jerbi, 2022).

Some limitations are to be noted regarding the present study. First, the sample size was limited, due to the rarity of participants with such extensive meditation abilities, thus possibly hampering the robustness of our findings. In addition, elderly participants in our cohort feature more meditation hours, thus our findings concerning correlation between complexity measures and expertise is partially confounded by age. It would thus be relevant to replicate this study with elderly novices to further corroborate our findings. Also, the cross-sectional nature of the study precluded strong conclusions about the effect of meditation experience on brain criticality. Another possible limitation concerns the calculation of LZC. In fact, if it is calculated on sequences of different lengths, the result may be influenced by changes in oscillatory frequency/duration of microstates. This is because if microstates have longer durations, there will be fewer transitions, and therefore complexity will be calculated on different length sequences.

## Data availability statement

The data analyzed in this study is subject to the following licenses/restrictions: data used in this study are protected as they might reveal confidential information about the participants. Nevertheless, data can be made available by the corresponding author upon reasonable request. Requests to access these datasets should be directed to laura.marzetti@unich.it.

## Ethics statement

The studies involving humans were approved by Local Ethics Commitee University of Chieti-Pescara. The studies were conducted in accordance with the local legislation and institutional requirements. The participants provided their written informed consent to participate in this study.

## Author contributions

AD’A: Conceptualization, Methodology, Visualization, Writing – original draft. PC: Formal analysis, Methodology, Visualization, Writing – original draft. JO’B: Methodology, Writing – original draft. KJ: Writing – original draft. AP: Writing – original draft. AR: Writing – original draft. VP: Conceptualization, Investigation, Writing – original draft. LM: Conceptualization, Funding acquisition, Supervision, Writing – original draft.
