# Supplementary material for: Mindfulness meditation styles differently modulate source-level MEG microstate dynamics and complexity
Source: Front Neurosci. 2024 Feb 2;18:1295615. doi: 10.3389/fnins.2024.1295615 (PMC10869546; doi:10.3389/fnins.2024.1295615)
Supplement: Supplementary file 1 [file Data_Sheet_1.docx]

***Supplementary Material***

**1 Supplementary figures**


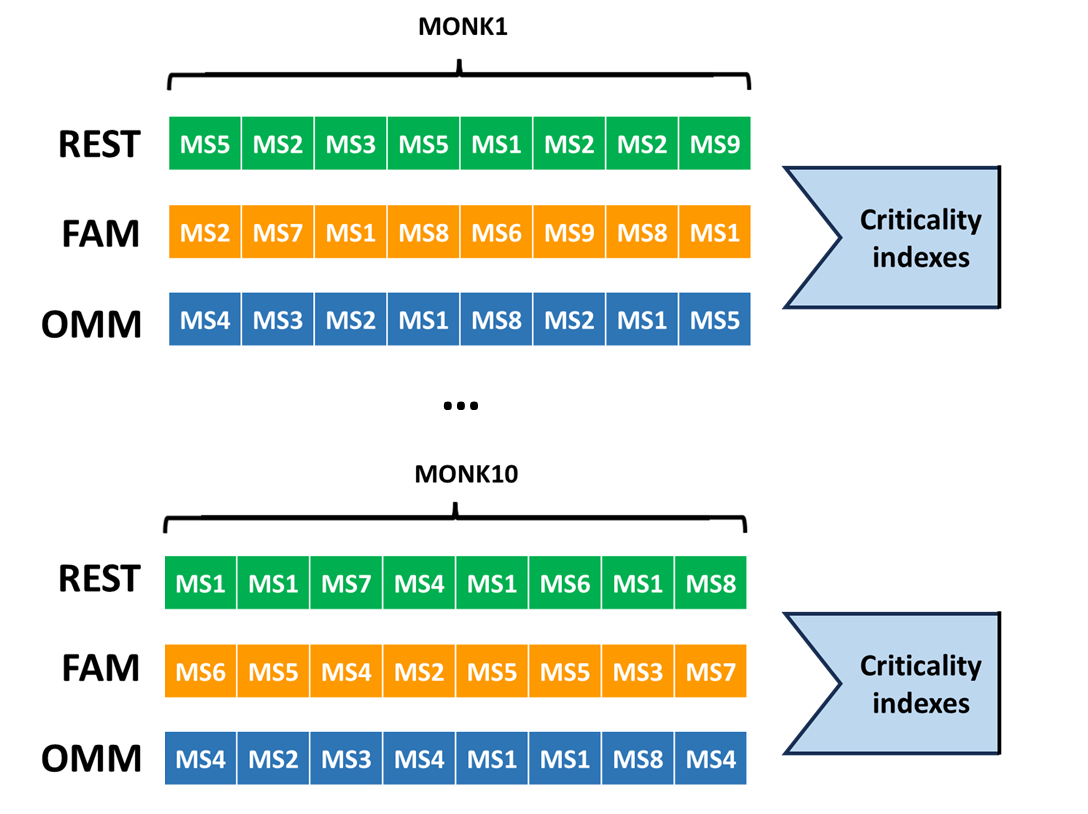


**Figure SM1. Schematic representation of microstate sequences in the different conditions.** In each condition, the temporal sequence of microstates obtained from each subject is fed to the algorithms for the computation of criticality indexes, i.e., Hurst exponent and Lempel-Ziv complexity.


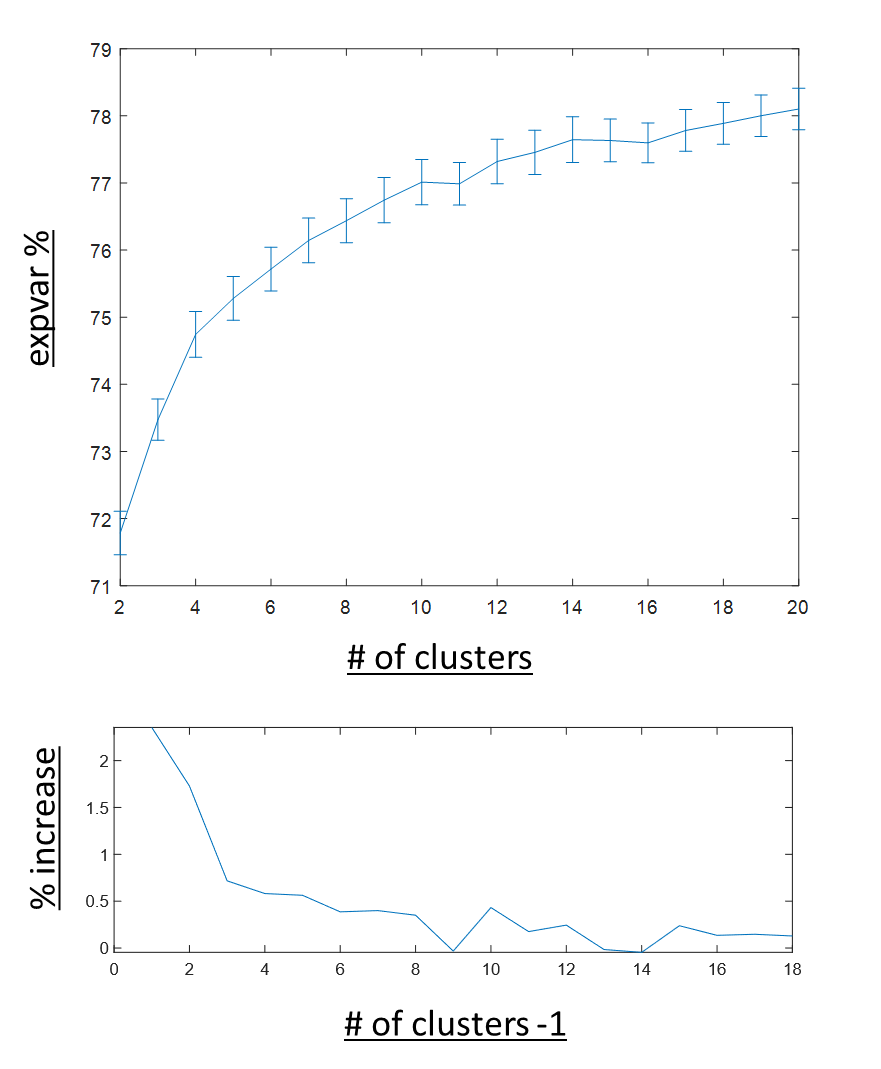


**Figure SM2. A. Explained variance as a function of the number of clusters. B. Percentage increase in explained variance as the number of clusters varies.**

**
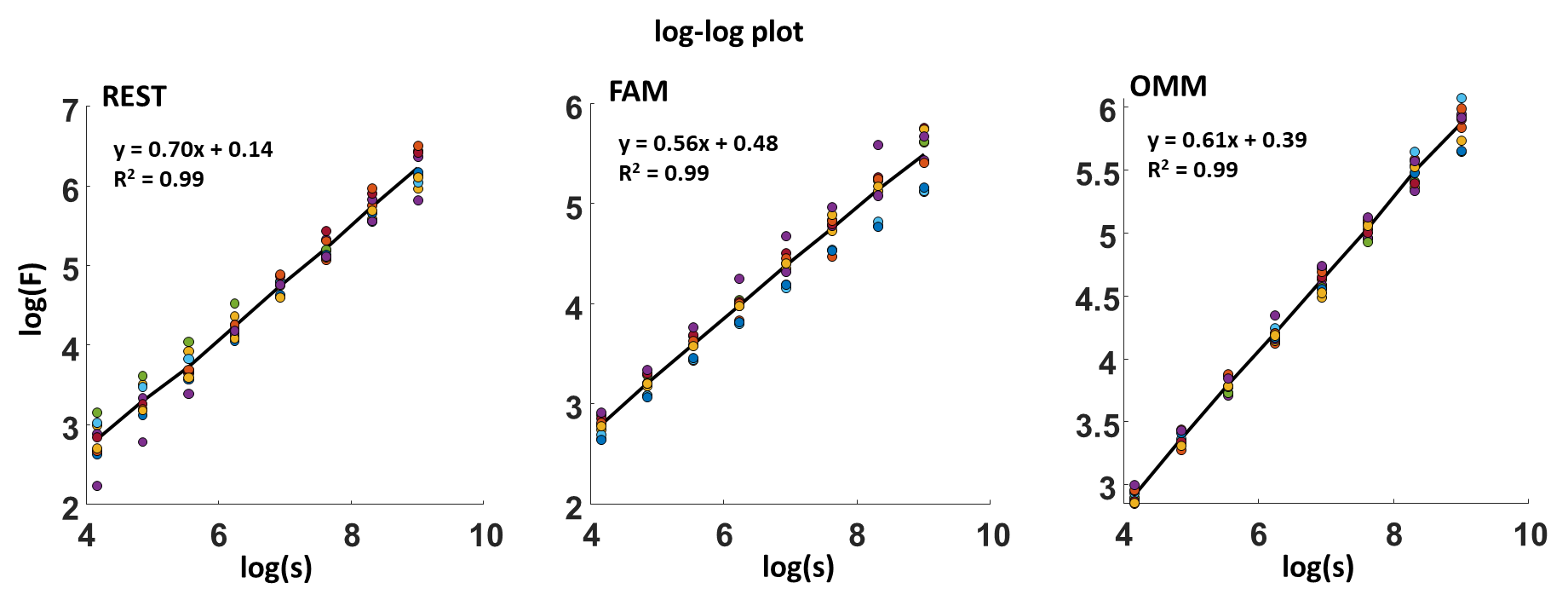
**

**Figure SM3. Log-log plot of the fluctuation F as a function of the window size s for each of the three conditions (REST, FAM, OMM).** The black line represents the mean of the fluctuation F across subjects, while the points depict the F values for each individual. Linear fit equations are also reported.

**Figure SM4.**

**2 Supplementary text**

The specific instructions given to participants before the experiment are reported below.

Focused Attention Meditation: “*Gently engage in sustaining the focus of your attention on breath sensations, such as at the nostrils, noticing with acceptance and tolerance any arising distraction, as toward stimuli or thoughts, and return gently to focus attention on the breath sensations after having noticed the distraction source*”.

Open Monitoring Meditation: “*Observe and recognize any experiential or mental content as it arises from moment to moment, without restrictions and judgment, including breath and body sensations, percepts of external stimuli, arising thoughts and feelings*''.
